# A Primer on Regression Methods for Decoding *cis*-Regulatory Logic

**DOI:** 10.1371/journal.pcbi.1000269

**Published:** 2009-01-30

**Authors:** Debopriya Das, Matteo Pellegrini, Joe W. Gray

**Affiliations:** 1 Life Sciences Division, Ernest O. Lawrence Berkeley National Laboratory, Berkeley, California, United States of America; 2 Department of Molecular, Cell, and Developmental Biology, University of California Los Angeles, Los Angeles, California, United States of America; 3 Comprehensive Cancer Center, University of California San Francisco, San Francisco, California, United States of America; Whitehead Institute, United States of America

## Introduction

### Importance of *cis*-Regulatory Elements

**Figure 1:**
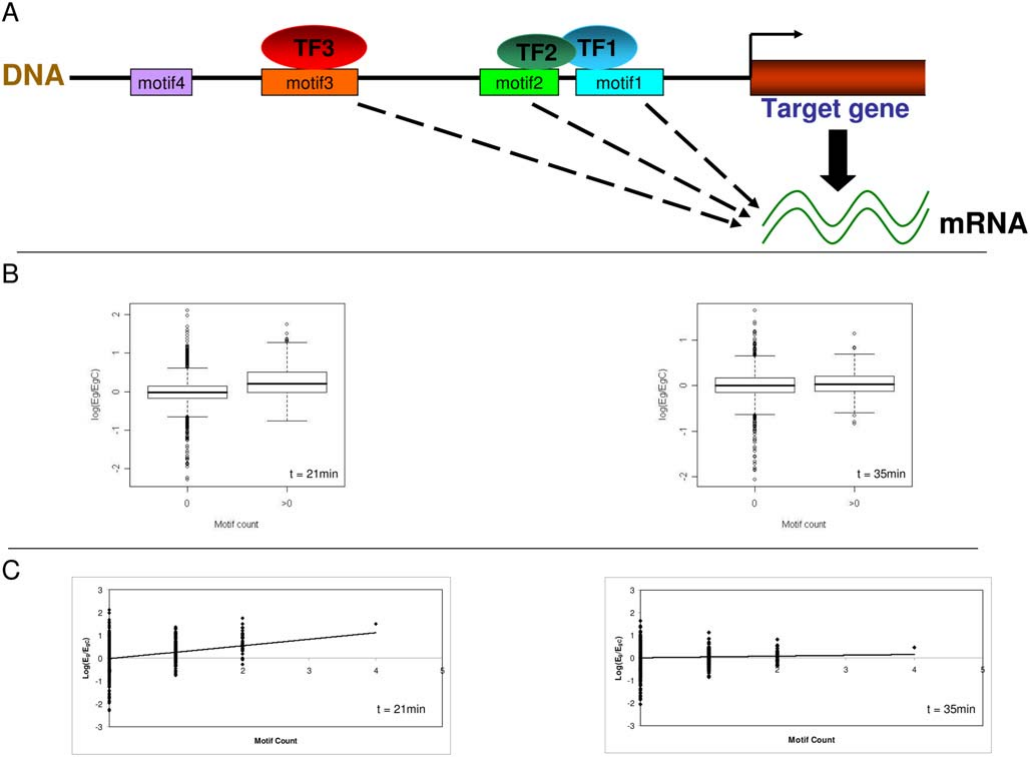
Basic Tenets of Modeling *cis*-Regulation Using a Regression Approach. (A) A schematic of transcriptional regulation is shown. Motifs 1, 2, and 3 are bound by their respective TFs and thus are active, while motif 4 is not. Furthermore, TFs 1 and 2 are shown to be interacting. (B) Box plots of the logarithm of expression ratio (E_g_/E_gC_) of genes containing the MCB element ACGCGT (marked as >0, group 1) and genes that do not contain the element (marked as 0, group 2) are shown for the alpha arrest experiment [8] of yeast cell-cycle. The ratio E_g_/E_gC_ is the expression of the gene relative to its average across all time points. During 21 min (G1/S phase), there is a statistically significant difference (*p*<1.0e-16, t-test) in expression level between the genes in groups 1 and 2. Average log_2_(E_g_/E_gC_) of these two groups is 0.27 and −0.02, respectively. During the 35 min (G2/M phase), there is no such association (*p* = 0.02, average log_2_(E_g_/E_gC_) = 0.04 vs 0.01). This type of approach is elucidated in detail in [57]. (C) The same data as in (B) is shown, except that the motif counts are no longer binary. There is a statistically significant association between the motif count and expression during the 21 min (*p* = 3.3e-12 (F-test), y = −0.02+0.28x), but not during the 35 min (*p* = 0.006, y = 0.01+0.04x) time point. Each point in the figure represents a gene, characterized by a count of ACGCGT in its promoter (*x*-axis) and log_2_(expression ratio) (*y*-axis).

The rapidly emerging field of systems biology is helping us to understand the molecular determinants of phenotype on a genomic scale [1]. *Cis*-regulatory elements are major sequence-based determinants of biological processes in cells and tissues [2]. For instance, during transcriptional regulation, transcription factors (TFs) bind to very specific regions on the promoter DNA [2],[3] and recruit the basal transcriptional machinery, which ultimately initiates mRNA transcription (Figure 1A).

### Learning *cis*-Regulatory Elements from Omics Data

A vast amount of work over the past decade has shown that omics data can be used to learn *cis*-regulatory logic on a genome-wide scale [4]–[6]—in particular, by integrating sequence data with mRNA expression profiles. The most popular approach has been to identify over-represented motifs in promoters of genes that are coexpressed [4],[7],[8]. Though widely used, such an approach can be limiting for a variety of reasons. First, the combinatorial nature of gene regulation is difficult to explicitly model in this framework. Moreover, in many applications of this approach, expression data from multiple conditions are necessary to obtain reliable predictions. This can potentially limit the use of this method to only large data sets [9]. Although these methods can be adapted to analyze mRNA expression data from a pair of biological conditions, such comparisons are often confounded by the fact that primary and secondary response genes are clustered together—whereas only the primary response genes are expected to contain the functional motifs [10].

A set of approaches based on regression has been developed to overcome the above limitations [11]–[32]. These approaches have their foundations in certain biophysical aspects of gene regulation [26], [33]–[35]. That is, the models are motivated by the expected transcriptional response of genes due to the binding of TFs to their promoters. While such methods have gathered popularity in the computational domain, they remain largely obscure to the broader biology community. The purpose of this tutorial is to bridge this gap. We will focus on transcriptional regulation to introduce the concepts. However, these techniques may be applied to other regulatory processes. We will consider only eukaryotes in this tutorial.

## Regression Methods for Learning the Active *cis*-Regulatory Elements

### What is a Regression Method?

A regression method is essentially a curve-fitting approach. When there is one observed variable (*y*-axis) and one predictor variable (*x*-axis), regression consists of drawing a line or a curve that best fits the data. The challenge arises when there are multiple candidate predictors, among which only a selected few are relevant. This is the case for *cis*-regulation, where relatively few *cis*-elements are differentially activated between two conditions while the number of candidate elements is large [2],[5]. Regression methods provide efficient ways to select this set of active elements via a curve-fitting exercise.

### How To Learn Which *cis*-Regulatory Elements Are Active

Let us consider the case of a single *cis*-element, a DNA word. Before we introduce the regression method, let us first proceed by dividing the genes into two groups, according to whether a gene has the word in its promoter or not. If under a biological condition the expression levels of genes in these two groups are significantly different from each other, it implies that the *cis*-element is most likely bound by its cognate TF, which is regulating its target genes. In other words, the *cis*-element is active. However, if there is no significant difference in expression between these two groups, then, analogously, the *cis*-element is likely inactive. Furthermore, if the genes with the *cis*-motif have higher expression levels on average than those without the motif, then the TF is an activator, and in the reverse situation an inhibitor. The case of the MCB element, a G1/S regulator of the yeast cell-cycle [8], is illustrated in Figure 1B. We observe that there is indeed a statistically significant association between the presence of the MCB element and mRNA expression in the G1/S phase of the cell-cycle (*p*<1.0e-16), but not in the G2/M phase (*p* = 0.02). Furthermore, this analysis indicates that the MCB element has an activating role in the G1/S phase, as expected [8].

A regression approach is a generalized version of the method described above. Here, the data is not binary any more. Instead, we plot the actual motif counts against the mRNA levels for all genes genome-wide (Figure 1C). To examine if there is any association between the occurrence of the MCB element and mRNA expression, we fit a straight line through these data points. Next, we check if the observed linear fit to the data could be obtained by random chance. If the fit is statistically significant, then the motif is considered active, just as in the binary data above, and inactive otherwise. Furthermore, if the slope of the fitted line is positive, then the element is an activator—a high number of elements are indicative of high expression on average, while fewer or no copies imply low expression. For the MCB element (Figure 1C), we notice that the fit is significant in the G1/S phase, but not in the G2/M phase, as expected of a G1/S-specific element. The positive slope of the line indicates that the MCB element is an activator.

The best fit shown in Figure 1C leads to a direct quantitative relation between the logarithm of observed expression *E_g_* and motif count *n_g_* of any gene *g* [11]:
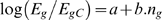
(1)where *C* indicates a reference condition. The parameters *a* and *b*, the intercept and slope of the line, respectively, are estimated from the input data via a least squares fit. *a* and *b* are constant across all genes. We can use Equation 1 to estimate how much of the mRNA expression levels are explained by this motif. We note that expression data from one experimental condition and one control condition are used in this analysis.

### How To Learn Multiple *cis*-Regulatory Elements

**Figure 2:**
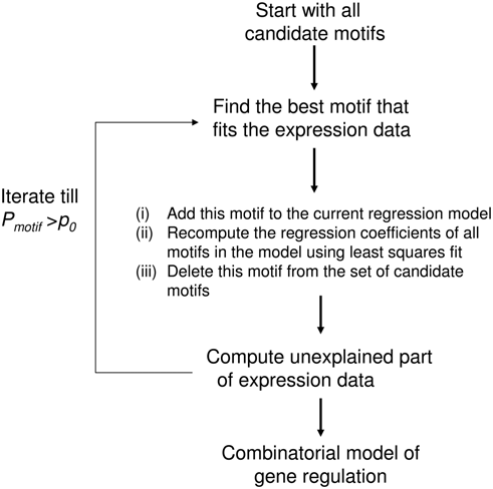
A Flow Chart for Modeling Combinatorial *cis*-Regulation Using Regression Methods. The steps are shown for constructing a model with linear functions; however, with some small modifications, they are applicable to nonlinear functions as well. *P_motif_* indicates the *p*-value of the association of the best motif with mRNA expression. *P_motif_*>*p_0_* is one possible termination condition. Other alternative strategies can also be used instead. In this example, feature selection and model building are done simultaneously.

Under any specific condition, multiple *cis*-elements are usually active [2],[36],[37]. Moreover, *cis*-regulation has been shown to be inherently combinatorial. Thus, often distinct combinations of such elements regulate the genes. To learn which specific combinations are active out of the many possible candidate elements, the simplest strategy is to repeat the above curve-fitting procedure for each such element. The elements that meet a significance threshold are considered to be active. However, this simple approach does not account for combinatorial regulation. Namely, it does not specify which particular elements act collectively to regulate gene expression. To overcome this limitation, we build a multivariate model (Equation 2 below with *d*_12_ = 0). This involves two steps: (a) feature selection, i.e., identifying which specific elements are active, and (b) model building, i.e., specifying the regression model involving these elements. These two steps may be executed simultaneously [11]. Alternatively, one can first select the *cis*-element features, and then build a regression model using these features [13]. A representative flowchart for multivariate modeling is shown in Figure 2. The elements that appear in a multivariate model are, then, hypothesized to be functional [11],[13].

An additional complexity is that functional interactions among TFs are often essential to transcriptional control [2]. This is especially true in higher organisms. In regression models, we introduce the interactions via a product of word counts. This reflects the fact that a pair of elements has a stronger effect than the sum of the elements in the pair. The strategy for including these terms is similar to the methodology described above [12]. For example, to describe the three motifs and interactions between motifs 1 and 2 in Figure 1A, the equation would be [12]:
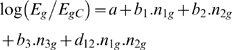
(2)
*n_ig_* is the count of motif *i* for gene *g*. The parameters *a*, *b*_1_, *b*_2_, *b*_3_, and *d*_12_ are learnt from the data, again using a least squares fit. *d*_12_>0 implies a synergistic interaction, while *d*_12_<0, a competitive interaction.

### How To Model Regulation by Degenerate Motifs

**Figure 3:**
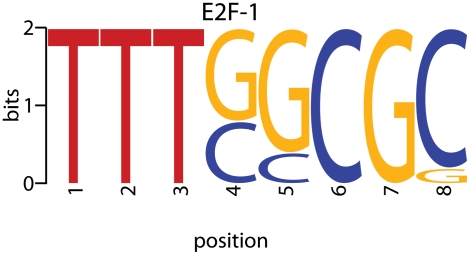
Position Weight Matrix (PWM) Logo for E2F-1. The sequence logo for the PWM of E2F-1, a key transcription factor for regulating the mammalian cell-cycle, is shown (http://jaspar.genereg.net/). The figure shows the bases that may occur at each position of this 8-nucleotide long motif. The height of each base quantifies the bits of information content, which is related to the probability of its occurrence at that position [3]. For example, there is a 100% chance of observing a T at position 1, while at position 8, a 90% chance of observing a C, and a 10% of G.

*cis*-Regulatory elements are often not simple words, especially in higher eukaryotes. Instead, the *cis*-elements bound by a specific TF may have small differences in their sequences in different promoters [4]–[6]. This variability, referred to as degeneracy of the motifs, is often represented by a position weight matrix (PWM) [3],[5]. PWMs are probabilistic representations of *cis*-motifs (Figure 3).

To use PWMs in regression methods, we would first score each promoter sequence against each PWM. The probabilities of each base at each position are used to compute the scores. These scores are related to the binding affinity of a TF for the DNA sequence [3],[35],[38]. There are multiple scoring schemes available 13,18,22,33 (see also [3],[35],[39]), but often the maximum score of a PWM for each promoter is used. We then use the same regression methods described above to construct a model, but with PWM scores instead of word counts. JASPAR [40] and TRANSFAC [41],[42] are among the most popular databases of PWMs. However, PWMs may also be generated using de novo motif discovery tools [4],[13],[43].

#### Nonlinear models

**Figure 4:**
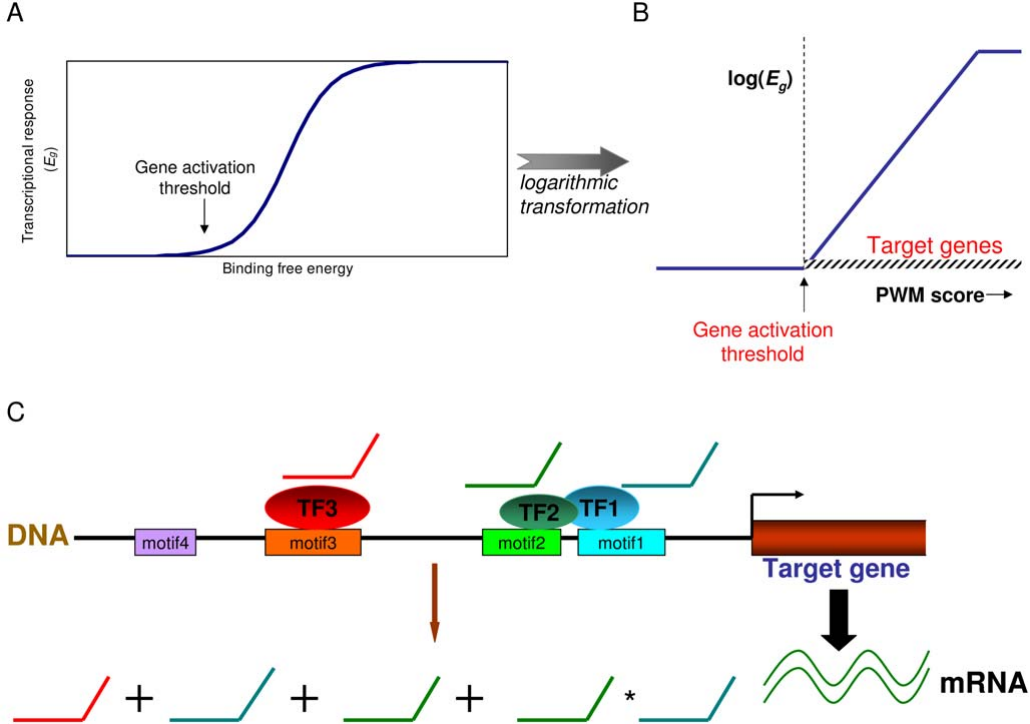
Nonlinear Regression Models of *cis*-Regulation. (A) mRNA expression (*E_g_*) as a function of TF binding free energy often has a sigmoidal pattern. There is an activation threshold, below which the transcriptional response is flat. Above the threshold, it grows exponentially, and finally saturates. For an inhibitory pattern, the curve is inverted along the *y*-axis. PWM scores are proportional to the binding free energies. (B) A logarithmic transformation of the sigmoidal function leads to a sum of linear splines. Each linear spline function has the shape of a hockey stick: It is zero below (or above) a threshold, called knot, and rises linearly above (or below) it. The smoothness of the transition from the flat part to the exponential part of the curve is not modeled in linear splines. A linear model is realized if the activation threshold is ignored, i.e., the sigmoidal function is replaced by an exponential function in (A). In a linear spline approach, the target determination threshold is set to the knot [22] or the gene activation threshold. While for the sigmoidal function, the threshold is typically set by the point at which the curve reaches half its maximal value [28]. (C) A model comprising linear splines for three functional motifs and one interacting motif pair is shown.

Although one can use linear methods with PWM scores [13], such methods are not ideal since the relation between motif scores and gene expression is not always linear. Furthermore, previous studies indicate that linear methods may not be optimal for modeling degenerate motifs when interactions are included [11]. This is a significant limitation since interactions among degenerate motifs are pervasive in mammalian transcriptional regulation [2],[5]. Instead, based on biophysical models, we expect the transcriptional response to be sigmoidal [44],[45] (Figure 4A). To account for such complexities, nonlinear methods have been developed. We model the expression ratios in terms of sums of sigmoidal functions of PWM scores [28],[31], or, alternatively, their variants, linear splines [15],[22]. Linear splines are related to sigmoidal functions by a logarithmic transformation (Figure 4B). They allow more efficient modeling when data is sparse since they require fewer parameters, while sigmoidal functions yield a more accurate model when sufficient data is available. The modeling procedure is similar to multivariate linear regression (see above). For the example shown in Figure 4C, we obtain an equation of the form:
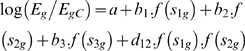
(3)Here, *s* denotes the PWM score. 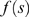 is a linear spline function or a sigmoidal function in *s*. Because of the increased number of fitting parameters, these more complex models require that we control for overfitting of the data. Although the implementation details are beyond the scope of this tutorial, they involve various forms of cross-validation (see the references in Table 1). These overfitting effects can also be significant in multivariate linear models with interactions. Because PWM scores are related to binding affinities, and sigmoidal functions model the essential biophysics of transcriptional regulation, these nonlinear approaches have strong biophysical underpinnings [26],[33],[34],[46].

**Table 1:** Regression Tools for *cis*-Regulatory Element Identification Currently Reported in the Literature.

Software/Publication	Reference	Linear or Nonlinear?	Degenerate or Nondegenerate Motifs?	Identifies Target Genes?	Web Site for Download
REDUCE	[11]	Linear	Nondegenerate	N	http://bussemaker.bio.columbia.edu:8080/reduce/
MODEM	[17]	Linear	Weakly degenerate	Y	http://wanglab.ucsd.edu/
Pham et al.*	[28]	Nonlinear (sigmoidal)	—	Y	NA
MARSMotif	[15]	Nonlinear (MARS)	Nondegenerate or weakly degenerate	N	http://rulai.cshl.edu/licensing/index1.htm
MARSMotif-M	[22]	Nonlinear (Linear spline/ MARS)	Degenerate	Y	http://rulai.cshl.edu/licensing/index1.htm
MotifRegressor	[13]	Linear	Degenerate	N	http://www.math.umass.edu/˜conlon/mr.html
Keles et al.	[12]	Linear	Nondegenerate	N	Available upon request
Motif Expression Decomposition (MED)	[23]	Nonlinear	Degenerate	Y	NA
Inferelator*	[24]	Nonlinear (LARS/LASSO)	—	Y	http://err.bio.nyu.edu/inferelator/
RSIR	[18]	Nonlinear (SIR)	Degenerate	N	Available upon request
MatrixREDUCE	[21]	Linear	Degenerate	N	http://bussemaker.bio.columbia.edu/software/MatrixREDUCE/
TRANSMODIS	[32]	Linear	Degenerate	Y	http://haedi.ucsd.edu/
Segal et al.	[31]	Nonlinear (sigmoidal)	Degenerate	Y	NA
Prego	[25]	Nonparametric	Degenerate	Y	http://uqbar.rockefeller.edu/˜atanay/prego/
MA-Networker*	[16]	Linear	—	Y	http://bussemaker.bio.columbia.edu/tools/
fREDUCE	[30]	Linear	Degenerate	N	http://genome3.ucsf.edu:8080/freduce/
SCAD	[29]	Nonlinear	Degenerate	N	NA

*: The tools marked with an asterisk were not originally used with *cis*-regulatory motifs as input, but can be easily adapted for this purpose.

NA indicates not available (we did not find this reported in the original paper or via Web search).

### How To Identify Target Genes

In a regression method, the input is a candidate motif. Thus, once we have identified the active motif, we have an additional task of determining which genes are targets of the cognate TF. Thus, in contrast to coexpression-based approaches where we assume that groups of co-expressed genes are co-regulated, co-regulation of genes is inferred in this approach a posteriori in regression methods. In the case of DNA words, it may seem that all promoters containing an instance of the word will always be bound by its partner TF. However, such a word may represent only the core of the motif. Thus, to discriminate the true targets, additional sequence information flanking the core motif may be essential [17],[32].

The challenge with the PWM scores is that they are generally continuous and nonzero (on a scale from zero to one, zero indicating that the motif is absent). Thus, most promoters often contain a low-scoring instance of each PWM. This is especially true for motifs of high degeneracy, as in humans [5]. Nonlinear regression methods provide a straightforward solution to select which instances of the motifs are active, since they allow one to define a cutoff threshold [22],[28] for each motif—promoters scoring above the threshold are then the targets, while those below are not (Figure 4B). There are alternative strategies to target determination, which are either more complex [23],[24],[31] or require information from ChIP-chip data [16],[25].

### How To Assess the Statistical Significance of the Fit

A popular metric to assess the quality of a regression model is how much of the variation in the expression data it can explain. This is parameterized as *R* ^2^, sometimes referred to as the percent reduction in variance [11]:
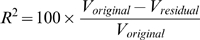
(4)where *V_original_* is the variance in the input expression data, and *V_residual_* is the variance of the differences between the input expression data and the fitted model. *V_residual_* represents the unexplained part of the variation in expression data. *R^2^* is directly related to the *F*-statistic [47], which is often used to evaluate the significance of the fit.

### Validity of the Premises

A large number of studies have shown that the motifs identified by regression methods are indeed functional motifs. The organisms where these methods have been applied include yeast [11]–[13], [15]–[18], [20], [21], [23], [25], [28]–[30],[32],[48], *C. Elegans* [32], *Drosophila* [14],[31], and human [22],[30]. Some of this work has been previously reviewed [26],[34],[49], and we refer to these publications for details.

### Which Kinds of Problems Can These Methods Be Applied to?

In this tutorial, we have focused on transcriptional regulation. However, regression methods may also be applied to other stages of gene regulation that are mediated by *cis*-elements. Regression approaches have been used to model chromatin remodeling [28], 3′ UTR mediated mRNA stability [50], and the regulation of alternative splicing of pre-mRNAs[27]. These methods can also be applied to DNA binding data, such as those generated by ChIP-chip [16],[51], DamID [14], or PBM [21],[52] experiments. In these cases, the binding ratios from TF binding profiles may be used in place of either expression ratios or motif scores, depending on the application.

### Available Software Based on Regression Methods

We have summarized the currently available software based on regression along with their key features in Table 1. The basic aspects of a regression method can be easily implemented in R or MATLAB.

## Conclusion

In this tutorial, we have described the basic aspects of regression methods. These are complementary to alternative approaches for motif discovery, such as comparative genomics [53]–[55] or motif over-representation methods [4],[56]. In particular, regression methods are optimal for evaluating the activity of *cis*-elements among a set of candidate elements. They are better suited for modeling combinatorial regulation and nonlinear responses and are more closely tied to the biophysical models of transcriptional regulation. With some modifications, regression methods can also be adapted for de novo motif discovery [21],[25],[50]. Finally, although most regression methods are used to model the observed changes in gene expression between a pair of conditions, recently this methodology has been extended to include information from multiple conditions as well [29].
